# Sedges in the mist: A new species of *Lepidosperma* (Cyperaceae, Schoeneae) from the mountains of Tasmania

**DOI:** 10.3897/phytokeys.28.5592

**Published:** 2013-11-04

**Authors:** George T. Plunkett, Karen L. Wilson, Jeremy J. Bruhl

**Affiliations:** 1 Botany, School of Environmental and Rural Science, University of New England, Armidale NSW 2351; 2 National Herbarium of New South Wales, Royal Botanic Gardens and Domain Trust, Sydney, Mrs Macquaries Road, Sydney NSW 2000

**Keywords:** Lepidosperma inops, Lepidosperma monticola, species limits, phenetics, new species

## Abstract

The status of a putative new species of *Lepidosperma* from the mountains of south-western Tasmania, Australia, was investigated. Phenetic analysis (Flexible UPGMA Agglomerative Hierarchical Fusion and semi-strong hybrid multidimensional scaling) was conducted on a database derived from morphological and anatomical characters scored from herbarium material, culm anatomy slides and scanning electron micrographs of fruit. The results of the analysis support the recognition of a new species, here described as *Lepidosperma monticola* G.T.Plunkett & J.J.Bruhl. The distribution, habitat and conservation status are discussed.

## Introduction

*Lepidosperma* Labill. (Cyperaceae) is a genus of c. 75 described species (Barrett and Wilson 2012) and up to 230 species based on current estimates of undescribed taxa (Barrett 2012). Australia is the centre of diversity for *Lepidosperma*, which also occurs in southern China, South-east Asia, New Caledonia and New Zealand. Species of *Lepidosperma* often form important components in diverse habitats that include beach dunes, forest and woodland, sedgeland and alpine heath (Barrett 2013).

The present study was initiated following the inspection by GTP of several specimens that had been identified as *Lepidosperma inops* F.Muell. ex Rodway. Eight of the specimens, collected in south-western Tasmania, appeared morphologically and ecologically distinct from the rest of the specimens, which were collected in the east of the state and attributable to *Lepidosperma inops* sens. strict. We intuitively segregated them as a putative new species, referred to as *Lepidosperma* sp. Eldon Bluff (A.M. Buchanan 9981). Subsequent plant collections in Tasmania and a visit to the Tasmanian Herbarium (HO) further strengthened our initial impression.

The aims of the study were (1) to determine the status and limits of this putative new entity and (2) to investigate its resemblance to morphologically similar named species of *Lepidosperma*.

## Materials and methods

### Study material

A total of 25 specimens were used for the phenetic analysis (Table 1). *Lepidosperma curtisiae* K.L.Wilson & D.I. Morris, *Lepidosperma inops* and *Lepidosperma tortuosum* F. Muell. were sampled on the basis of morphological similarity to *Lepidosperma* sp. Eldon Bluff. Specimens recently collected in Tasmania and existing specimens from the N.C.W. Beadle Herbarium (NE) were used in the analysis. Specimens on loan from the National Herbarium of Victoria (MEL) and HO for a previous study (Hodgon et al. 2006) were also used, with permission.

**Table 1.** Specimens of *Lepidosperma* used in phenetic analysis. First three letters of operational taxonomic unit (OTU) code: cur = *Lepidosperma curtisiae*; eld = *Lepidosperma* sp. Eldon Bluff; ino = *Lepidosperma inops*; tor = *Lepidosperma tortuosum*. Localities are in Tasmania unless another State is specified.

OTU Code	Voucher
curBag01	Above Bagdad Rivulet, Pontville, *A.J. North* *s.n.*, 8 October 1996, HO 322622 (HO)
curCon01	Conara, Midlands, *J.J. Bruhl 3005* (NE)
curMac01	Macquarie River, 0.5 km SW of Long Marsh Dam, *A. Moscal 8040* (HO)
curRos01	Roses Creek Road, Grampians, Victoria, *A.C. Beauglehole 25084* (MEL)
eldCou01	Mt Counsel, South West, *S.J. Jarman* *s.n.*, 12 March 1986, HO 411078 (HO)
eldEld01	Below Eldon Bluff, Central Highlands, *A.M. Buchanan 9981* (HO)
eldFie01	Mt Field National Park, Tarn Shelf, *G.T. Plunkett 99* (NE)
eldFie02	Mt Field National Park, Tarn Shelf, *G.T. Plunkett 100* (NE)
eldMcC01	Mt McCall, West Coast, *S.J. Jarman* *s.n.*, HO 411443 (HO)
eldPro01	Mt Propsting, South West, *M.J. Brown 1365* (HO)
eldSpr01	Mt Sprent, South West, *G.T. Plunkett 94a* (NE)
eldSpr02	Mt Sprent, South West, *G.T. Plunkett 96* (NE)
eldSpr03	Mt Sprent, South West, *G.T. Plunkett 98* (NE)
inoBre01	Mills Marsh, Break-O-Day, East Coast, *A. Moscal 6182* (NE)
inoGra01	Grasstree Hill, East Coast, *A. Moscal 18031* (NE)
inoLen01	Lenah Valley, Midlands, *D.A. Ratkowsky 1083* (NE)
inoUni01	University of Tasmania, *G.T. Plunkett 112* (NE)
inoUni02	University of Tasmania, *G.T. Plunkett 115* (NE)
torBaro02	Barokee Swamp, Cathedral Rock National Park, Northern Tablelands, NSW, *J. Hodgon 415* (NE)
torBarT01	Barrington Tops National Park, Northern Tablelands, NSW, J.R. Hosking 3041 (NE)
torBla01	2 km W of Blackheath, Central Tablelands, NSW, V. *Klaphake* *s.n.*, 1 January 1998, NE 808939 (NE)
torBoo01	Boonoo Boonoo State Forest, Northern Tablelands, NSW, *J.J. Bruhl 2453* (NE)
torDem01	Demon Nature Reserve, Northern Tablelands, NSW, *J.T. Hunter 5068* (NE)
torMul01	Surveyors Creek Fire Trail, from Mulligans Drive end, Gibraltar Range National Park, Northern Tablelands, NSW, *J. Hodgon 391c* (NE)
torWer01	Werrikimbe National Park, Northern Tablelands, NSW, *S.J. Griffith* *s.n.*, 20 April 1993, NE 66428 (NE)

### Anatomy

Hand-cut cross-sections of culms were made in 50% ethanol using half a double edged razor blade from pickled (70% ethanol) or rehydrated herbarium material. Sections were stained in 1% safranin in 50% ethanol for c. 15 min then washed in increasing concentrations of ethanol before staining in 0.5% fast green in 95% ethanol for three to seven minutes depending on size. Sections were then transferred to increasing concentrations of histolene before mounting on glass slides with Eukitt mountant.

Photosynthetic pathway was inferred from anatomy using the ‘maximum cell distance count’ criterion as applied to Cyperaceae (Bruhl et al. 1987; Bruhl and Wilson 2007).

### Scanning electron microscopy

Where available, a single representative fruit in good condition was removed from each operational taxonomic unit (OTU). Additional fruit removed from non-OTU specimens were also used. Fruit were mounted onto an aluminium stub with double-sided carbon tabs and gold-coated using a JEOL MP-19020NCTR NeoCoater (JEOL Ltd., Tokyo). Images were captured with a JEOL JCM-5000 benchtop SEM (JEOL Ltd., Tokyo) at 10 kV. In total three fruit were imaged from *Lepidosperma curtisiae*, four from *Lepidosperma inops*, seven from *Lepidosperma* sp. Eldon Bluff and two from *Lepidosperma tortuosum*.

### Characters

A character list was constructed primarily from that used by Hodgon (2001) and augmented by Barrett (2007a, b). Further characters were added based on differences among specimens observed by the authors. A total of 131 morphological and 22 anatomical characters were scored for each individual specimen (Appendix 1).

Morphological characters were scored from herbarium specimens with the aid of a Leica M8 stereomicroscope. Morphological dimensions were measured once per OTU, consistently using a steel rule, electronic callipers or the microscope eyepiece graticule for each character. Fruit and perianth characters were assessed under the stereomicroscope and from SEM images. Anatomical characters were scored from permanent double-stained slides viewed under a Zeiss Axiolab compound microscope.

### Analysis

Data were organised using DELTA ver. 1.04 (Dallwitz 1980) and later transferred to PATN ver. 3.12 (Blatant Fabrications) for cluster and ordination analysis (Appendix 2). Characters with multiple states scored for several OTUs were converted to multiple binary characters. Univariate characters and those used only for descriptive purposes were removed prior to analysis, as were characters that had missing values for the majority of OTUs. All characters were weighted equally in the analysis. Phenograms were produced from classification analysis run using Flexible UPGMA Agglomerative Hierarchical Fusion (*β* = -0.1). Three-dimensional ordination plots of semi-strong hybrid multidimensional scaling (SSH MDS) were generated. Lowest stress in the ordination analysis was achieved with 400 random starts and 100 iterations. Principal component correlation (PCC) was used to determine the importance of characters in the ordination analysis.

Our species concept is consistent with that of de Queiroz (1998, 1999, 2007), which equates species with segments of separately evolving metapopulation lineages. Here we base the delimitation of species on phenetic distinguishability, ecological differentiation, and diagnosability. The presence of one of these properties provides evidence that a lineage is evolving separately and can be recognised as a species, and the presence of additional properties provides greater confidence (de Queiroz 2007). Operationally we use a morphological species concept combined with implicit use of the biological species concept (lack of gene flow and geographical separation) and an ecological species concept (most similar species occurring in distinctly different habitats).

In the phenetic analyses, our criteria for accepting the putative species as a distinct entity were: (1) the OTUs representing *Lepidosperma* sp. Eldon Bluff should form discrete groups distinct from all other groups of OTUs in the analyses and (2) the OTUs within these groups should show an amount of dissimilarity comparable to that of the known species included in the analyses.

## Results and discussion

### Phenetic analysis

**Figure 1.**
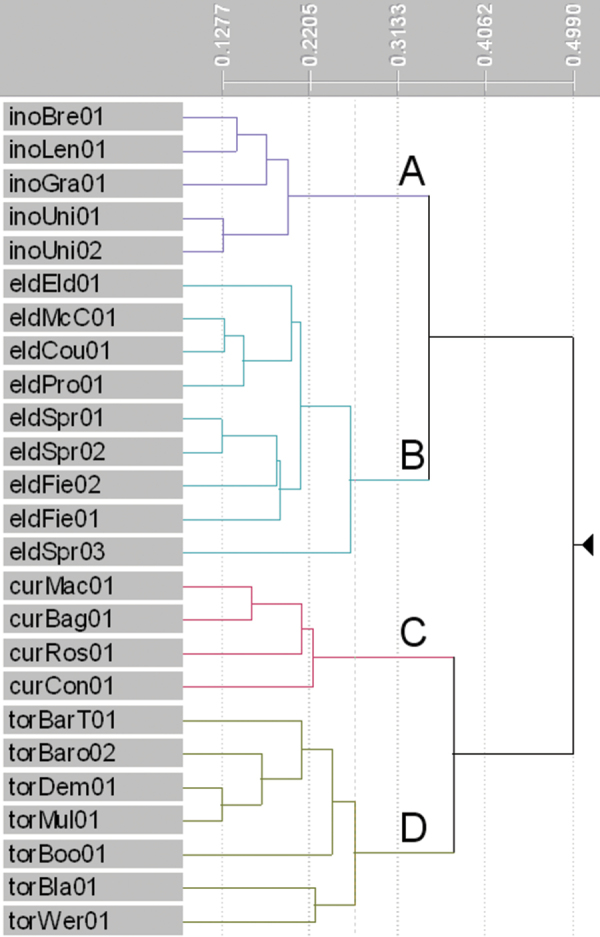
Phenogram produced from cluster analysis of three described and one putative species of *Lepidosperma* (*β* = -0.1). Major groups: **A** *Lepidosperma inops* (purple) **B** *Lepidosperma* sp. Eldon Bluff (turquoise) **C** *Lepidosperma curtisiae* (pink) **D** *Lepidosperma tortuosum* (green-grey). See Table 1 for operational taxonomic unit codes, Appendix 1 for characters and Appendix 2 for data.

The phenogram produced from the cluster analysis (Figure 1) shows distinct clusters of OTUs that match the three described and one putative species. The levels of dissimilarity within these four groups are comparable. Groups A and B in the phenogram contain individuals of *Lepidosperma inops* and *Lepidosperma* sp. Eldon Bluff, respectively. These groups are most similar to one another, as expected, though clearly distinct. The *Lepidosperma inops* group shows the lowest dissimilarity among individuals, possibly because of the lower number of OTUs used for that species. Group C contains individuals of *Lepidosperma curtisiae* and shows low dissimilarity among individuals, again possibly related to the low number of OTUs used. Group D contains individuals of *Lepidosperma tortuosum* and displays the highest dissimilarity among individuals, reflecting the morphological variation sampled in this species.

**Figure 2.**
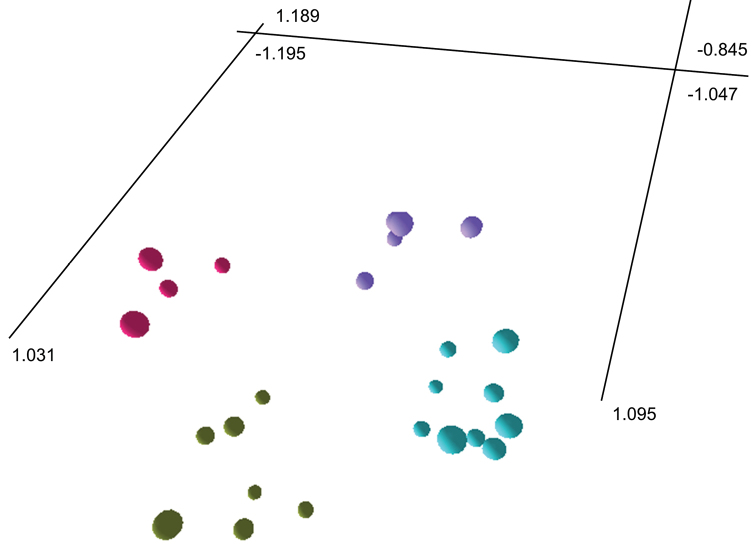
Three-dimensional ordination from semi-strong hybrid multidimenstional scaling of three described and one putative species of *Lepidosperma*. *Lepidosperma curtisiae* (top left; pink); *Lepidosperma inops* (top right; purple); *Lepidosperma* sp. Eldon Bluff (bottom right; turquoise); *Lepidosperma tortuosum* (bottom left; green-grey). Ordination oriented to highlight separation of groups of operational taxonomic units. See Appendix 1 for characters and Appendix 2 for data.

The ordination plot produced from SSH MDS (stress value 0.1535; Figure 2) recovered the same four groups as the phenogram. Individuals of *Lepidosperma curtisiae* and *Lepidosperma inops* respectively form the two most closely spaced groups, with individuals of *Lepidosperma tortuosum* forming the most distantly spaced group. Individuals of *Lepidosperma* sp. Eldon Bluff form a distinct group that is close to the group containing *Lepidosperma inops*.

The PCC values for the ordination analysis (Table 2) indicate that the most important characters were mostly vegetative and inflorescence morphology characters, with one fruit character and three anatomical characters.

**Table 2.** Principal component correlation of characters and ordination vectors from semi-strong hybrid multidimensional scaling ordination of three described and one putative species of *Lepidosperma*. Characters with a maximum correlation (*r^2^*) higher than 0.7 are included. See Appendix 1 for characters and Appendix 2 for data.

No.	Character	X	Y	Z	r^2^
128	Fruit style cap indumentum	-0.718	0.3	0.628	0.845
9	Culm margin hairs: orientation	0.645	-0.12	-0.755	0.834
38	Leaf lamina margin hairs: orientation	0.407	-0.667	-0.624	0.767
2	Culm length	0.129	0.18	-0.975	0.765
17	Angle of ramet	-0.643	0.34	0.686	0.74
145	Culm pith: homogeneous or heterogeneous	0.086	0.007	0.996	0.734
72	Rachis: whether reflexed	0.058	-0.243	0.968	0.725
62	Involucral bract ligule: emarginate	-0.11	-0.064	-0.992	0.721
153	Culm sub-epidermal fibres: depth	0.854	-0.4	-0.334	0.709
58	Involucral bract sheath: indumentum	-0.208	-0.971	0.114	0.707
136	Culm vascular bundles: number of size classes	-0.146	-0.382	-0.913	0.701

### Scanning electron microscopy

**Figure 3.**
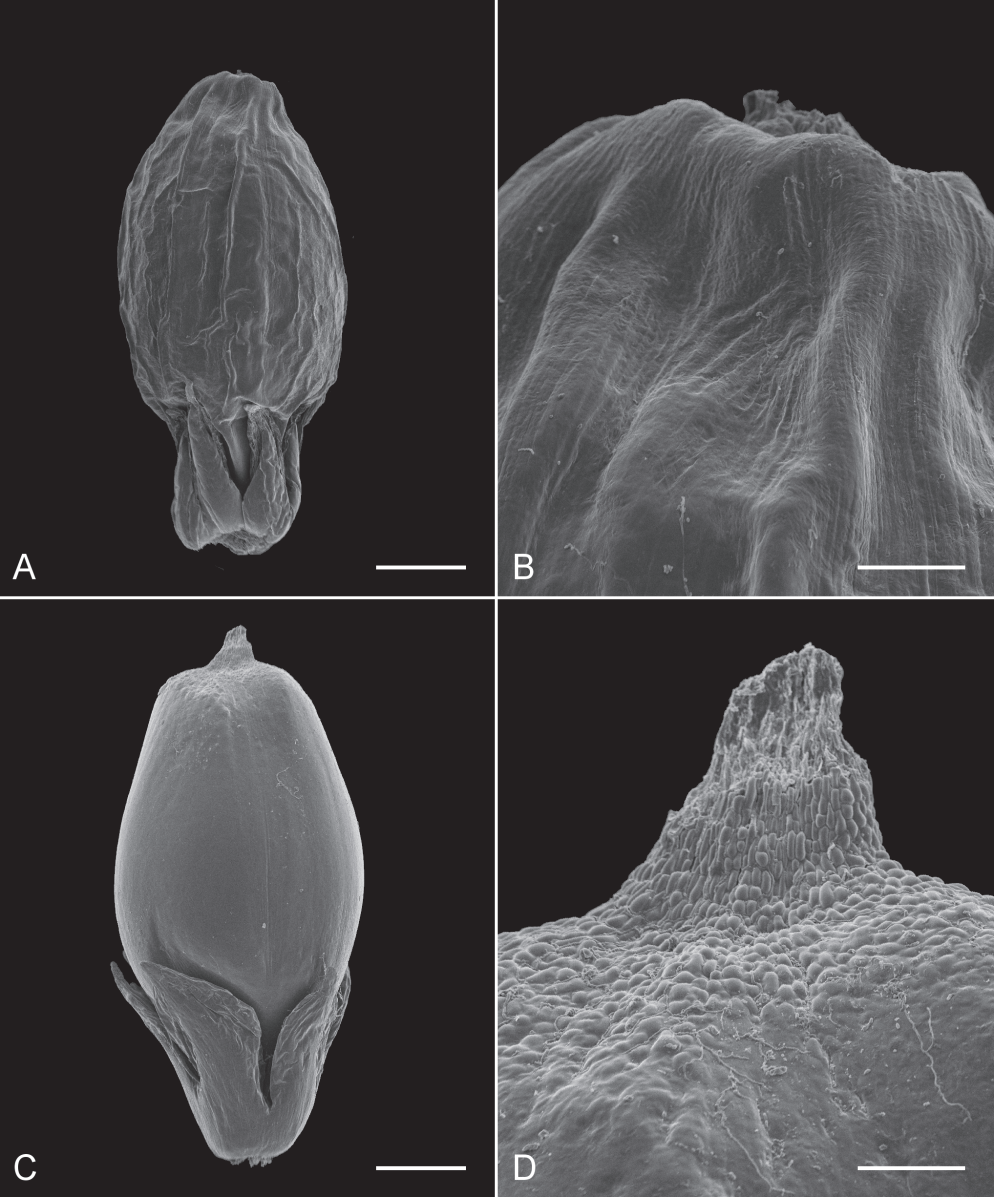
Scanning electron micrographs of fruit with perianth (**A, C**) and fruit apex (**B, D**). **A, B** *Lepidosperma inops* (*A. Moscal 18031*) **C, D** *Lepidosperma* sp. Eldon Bluff (*G.T. Plunkett 100*). Scale bars = 0.5 mm (**A, C**), 0.1 mm (**B, D**).

The images obtained with SEM demonstrated clear differences between fruits of *Lepidosperma* sp. Eldon Bluff and its closest neighbour *Lepidosperma inops* (Figure 3). The fruit of *Lepidosperma* sp. Eldon Bluff is minutely but distinctly colliculate at its distal end (Figure 3D), while the fruit of *Lepidosperma inops* is smooth distally (Figure 3B). The size and shape of the fruit and the size of the perianth varies between the two specimens shown here (Figure 3A, C), but no consistent differences were found in perianth size and morphology between the two taxa. Based on our observation of fruit from other species of *Lepidosperma*,the wrinkled fruit of *Lepidosperma inops* shown here, although well-developed, is slightly immature and the wrinkling is not diagnostic (old fruits on the same collection in NSW were too fragile to image but still intact enough to see that the main body is only very faintly wrinkled). More mature fruits of *Lepidosperma inops* suitable to image were not available. Fruits of *Lepidosperma curtisiae* and *Lepidosperma tortuosum* are also smooth distally (not shown here). Observations of additional fruit under the stereomicroscope were consistent with the SEM images.

## Taxonomy

**Figure 4.**
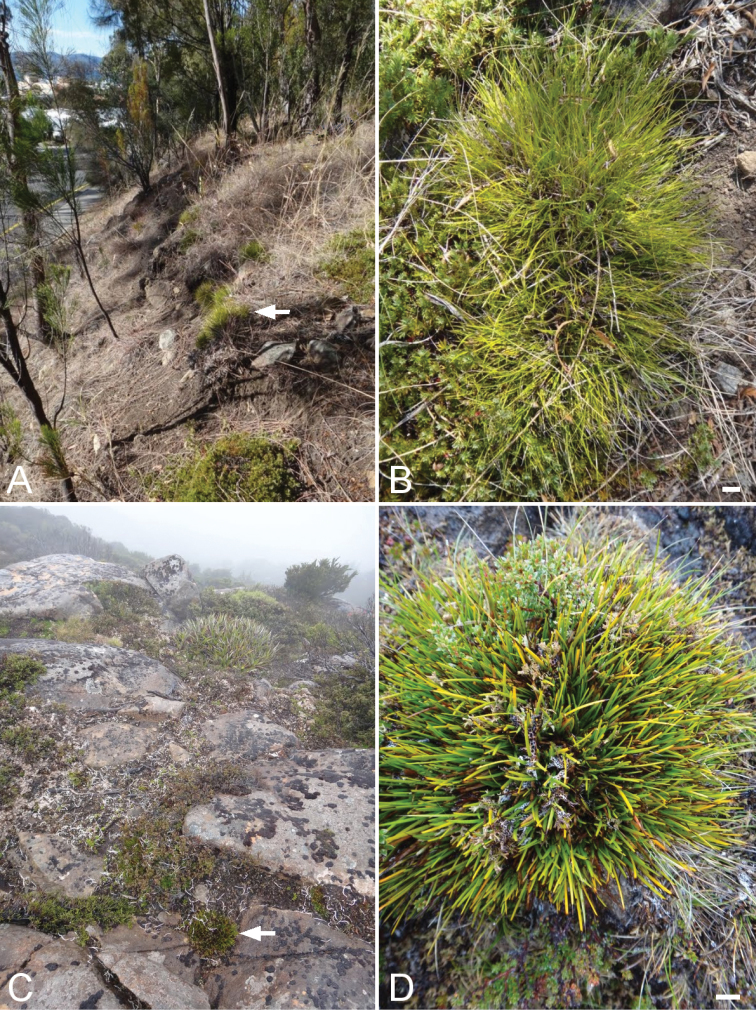
Comparison of habitat and habit of *Lepidosperma inops* and *Lepidosperma monticola*. **A, B** *Lepidosperma inops* (*G.T. Plunkett 112*) at University of Tasmania campus, Hobart **C, D** *Lepidosperma monticola* (*G.T. Plunkett 99, J.J. Bruhl & C.J. Prychid*) at the type locality, Tarn Shelf, Mt Field National Park. Scale bars = 1 cm. Arrows indicate plants of *Lepidosperma inops* (**A**) and *Lepidosperma monticola* (**C**).

The results of the phenetic analysis provide strong support for the acceptance of *Lepidosperma* sp. Eldon Bluff as a distinct entity. In both the phenogram (Figure 1) and ordination (Figure 2) a discrete group is formed by OTUs representing *Lepidosperma* sp. Eldon Bluff. This group shows a level of dissimilarity that is comparable to those of the known species of *Lepidosperma* included in the analysis. The SEM images also show that *Lepidosperma* sp. Eldon Bluff has distinctive fruit (Figure 3). This evidence provides strong support for the recognition of *Lepidosperma* sp. Eldon Bluff as a separate species from *Lepidosperma inops*. The ecological and geographic isolation of these two entities adds further support to this inference (Figure 4; Table 3). *Lepidosperma* sp. Eldon Bluff is described below as *Lepidosperma monticola* sp. nov.

**Table 3.** Selected morphological and ecological attributes separating *Lepidosperma monticola* and *Lepidosperma inops*. Ranges presented are absolute.

	*Lepidosperma inops*	*Lepidosperma monticola* sp. nov.
**Morphological character^**		
Culm length to leaf length ratio	0.27–0.37	0.53–0.7
Involucral bract length	27–59 mm	9–25 mm
Fruit – distal surface	smooth	minutely colliculate
Angle of ramet (i.e. between outermost leaves)	9–15°	20–27°
**Ecology**		
Habitat	Grassy woodland or sclerophyll forest	Alpine heath, herbfield, occasionally subalpine woodland †
Altitude	20–500 m	700–1170 m

^ Measurements from five specimens of *Lepidosperma inops* and nine of *Lepidosperma monticola*, fruit observations from four and seven specimens respectively.

† The vegetation at Eldon Bluff  for *A.M. Buchanan 9981* (HO) at 1080 m is described as “Open *Eucalyptus coccifera* forest on dolerite talus.”

### Lepidosperma monticola

G.T.Plunkett & J.J.Bruhl sp. nov.

urn:lsid:ipni.org:names:77133478-1

http://species-id.net/wiki/Lepidosperma_monticola

#### Remarks.

*Lepidosperma monticola* is distinguished from *Lepidosperma inops* in having a minutely colliculate fruit apex, its longest culms being greater than half the length of the leaves, and the angle between the outermost leaves of the ramets being 20° or greater.

#### Type.

**Figure 5.**
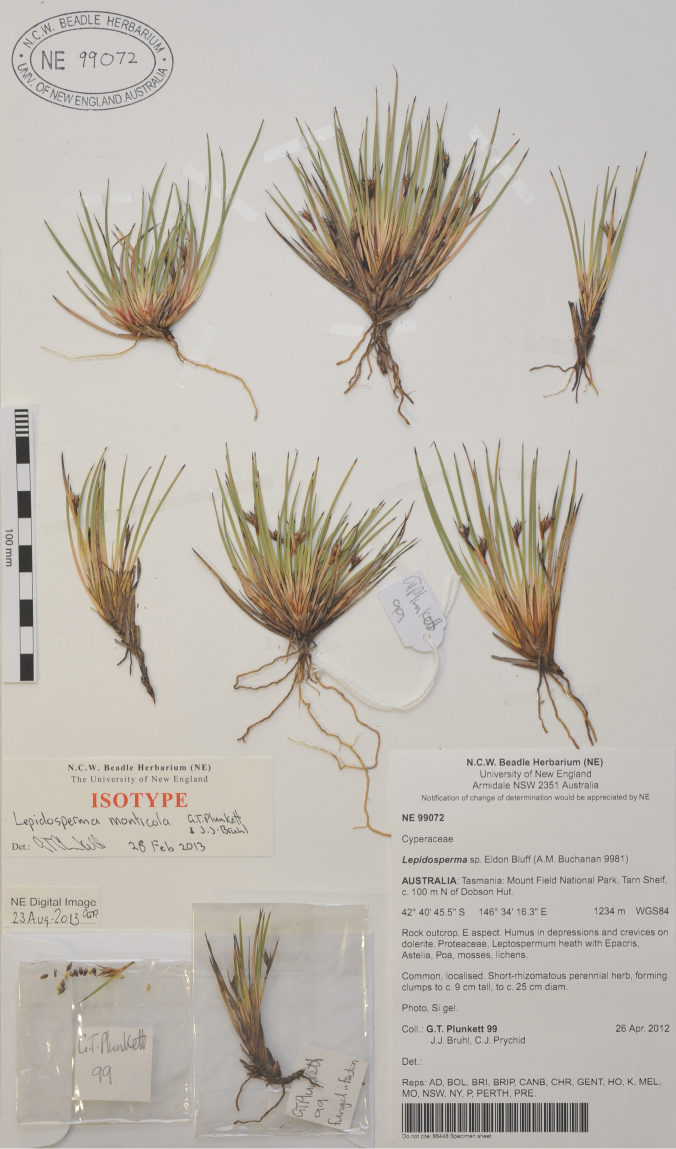
Isotype (*G.T. Plunkett 99* et al., NE) of *Lepidosperma monticola* G.T.Plunkett & J.J.Bruhl, sp. nov.

AUSTRALIA: Tasmania: Mt Field National Park, Tarn Shelf, c. 100 m N of Dobson Hut, 26 April 2012, *G.T. Plunkett 99, J.J. Bruhl & C.J. Prychid*; holotype: HO; isotype: AD, BOL, BRI, BRIP, CANB, CHR, GENT, K, MEL, MO, NE, NSW, NY, P, PERTH, PRE. (Figure 5).

*Short-rhizomatous perennial*, forming dense clumps. *Leaves* markedly distichous, up to 8 cm long, innovations forming flat fans (ramets) with angle of 20–27° between outer leaves; lamina well-developed, isobilateral, shallowly biconvex, ± rigid, striate, to 65 mm long, 1–1.5 mm wide, margins glabrous or scabrous with prickle hairs antrorse; sheaths 10–25 mm long, pale yellow-brown to mid-brown, often with tinge of red-pink, not resinous, margin glabrous; ligule subulate to acute, glabrous. *Culms* shallowly biconvex or rhombic in cross-section, 30–55 mm high, 0.8–1.4 mm wide, 1/2–2/3 length of longest leaves, pale yellow-brown at base, margins glabrous or scabrous with prickle hairs antrorse. *Photosynthetic pathway* ‘maximum cell distance count’ >1, C_3_.*Inflorescence* obovate in outline, a reduced panicle of 1–3 spikelets, 7–12 mm long, 2.5–5(–10) mm wide; involucral bract equal to or up to twice as long as the inflorescence, 9–24.5 mm long, sheath pink-red to dark red-brown at least proximally; rachis not flexous or reflexed. *Spikelets* 5–6.5 mm long, 1–2 mm diameter, with single bisexual flower; prophylls acute or emarginate, puberulous. *Glumes* 4, all of similar length; lowest 2 sterile, mucronate, outer face scabrous, with raised midrib; fertile glumes c. 6 mm long, apex subulate, midrib indistinct, outer face puberulous, margins glabrous. *Perianth scales* 6(–8), acute to acuminate, glabrous, 0.8–1.6 mm long. *Anthers* c. 1.4 mm long excluding apiculus; apiculus 0.4 mm long, glabrous. *Nut* elliptical in outline, pale green to mid brown depending on maturity, with 3 discolorous and raised ribs, c. 3 mm long, 1.4–1.7 mm diameter; style cap truncate, minutely colliculate.

#### Distribution and habitat.

**Figure 6.**
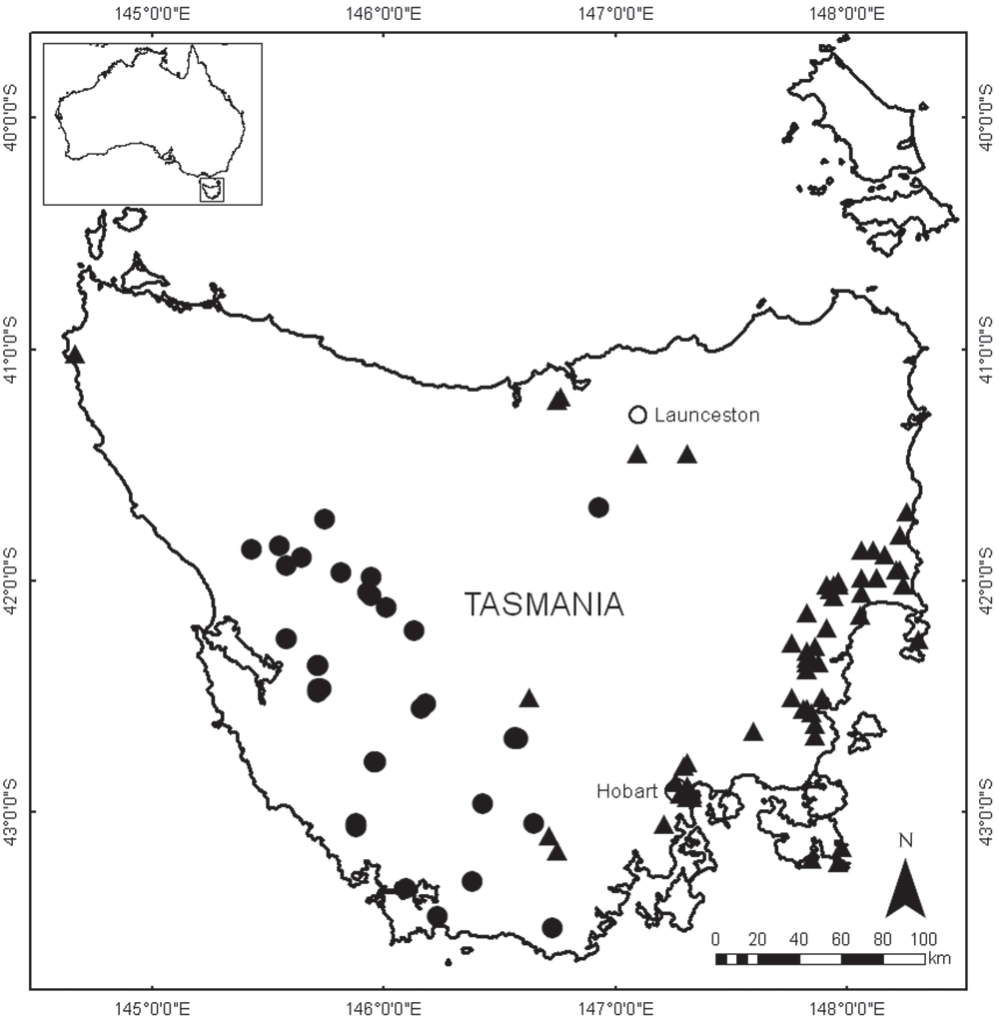
Distribution of *Lepidosperma inops* (solid black triangles) and *Lepidosperma monticola* (solid black circles) from herbarium specimen data. Cities indicated by open circles. All specimens seen by G.T. Plunkett (see Appendix 3 for vouchers).

Restricted to the South West, Central Highlands, West Coast and Mt Field regions of Tasmania; in alpine heath, herbfields, open forest and moorland at altitudes greater than about 700 m (Figure 6). At Tarn Shelf (Figure 4) and Mt Sprent this species grows in skeletal humus over or amongst rock outcrops, in epacrid–Proteaceae–Myrtaceae heath or *Gymnoschoenus sphaerocephalus* sedgeland.

#### Conservation status.

On the basis of our current knowledge, this species would not warrant listing under either the International Union for Conservation of Nature Red List (IUCN 2012), Australian *Environment Protection and Biodiversity Conservation Act* 1999 or Tasmanian *Threatened Species Protection Act* 1995. All populations known from herbarium material occur within National Parks, Regional Reserves or Conservation Areas.

#### Derivation of epithet.

Named from the Latin *mons,montis* (mountain) and -*cola* (dweller), referring to the distribution of this species on the mountains of Central and South Western Tasmania.

#### Selected specimens examined.

**AUSTRALIA. Tasmania: Central Highlands:** 3 km SE of Pyramid Mountain, 1100 m, 14 Feb. 1983, *A. Moscal 1776* (HO); Cradle plateau, 880 m, 7 Mar. 1949, *W.M. Curtis* *s.n.* (HO); High Dome, 24 Feb. 1994, *J.B. Kirkpatrick* *s.n.* (HO); Sticht Range, 20 km S of Tullah, 920 m, 16 Apr. 1990, *P.A. Collier 4678* (HO). **Mt Field:** Shelf above University Hut, Lake Dobson, 1120 m, 1 Jan. 1949, *W.M. Curtis* *s.n.* (HO). **South West:** Elliot Range, summit, 900 m, 15 Jan. 1985, *S.J. Jarman* *s.n.* (HO, MEL, NSW); Mt Rugby, 1120 m, 16 Feb. 1978, *S.J. Jarman* *s.n.* (HO). **West Coast:** Mt Darwin, 1100 m, 8 Mar 1974, *D.A. Ratkowsky* *s.n.* (HO); Mt Dundas, summit, 860 m, 1 Mar. 1894, *L. Rodway* *s.n.* (HO); Range extending S to SE from Mt Curly, 5 Feb. 1985, *S.J. Jarman 263* (HO).

#### Phenology.

Flowers November to February. Fruits December to April.

## Supplementary Material

XML Treatment for Lepidosperma monticola

## Appendix 1

Annotated character list used for phenetic analysis of species of *Lepidosperma* and description of *Lepidosperma monticola* sp. nov. Modified from DELTA ver. 1.04 database output.

**Vegetative morphology:**

#1. Rhizomes <whether long or short>/
  1. long <shoots spreading>/
  2. short <shoots clumped>/
  • If long and short, both states scored
#2. Culms <length including inflorescence>/
  mm long/
  • Measured from base of plant (i.e. base of leaf sheaths) to the most distal inflorescence spikelet
#3. Culms <width at mid-third>/
  mm wide/
  • The greatest dimension in elliptical/flattened culms. Measured in the mid-third of culm length
#4. Culms <thickness at mid-third>/
  mm thick/
#5. Culms width to thickness ratio <at mid-third>/
  • Longest culm measured
#6. Culms <when dried; whether striate>/
  1. striate/
  2. not striate/
#7. Culms <whether deeply grooved>/
  1. <deeply> grooved/
  2. not <deeply> grooved/
  • One or more deep grooves obvious at 10x magnification on fresh or dried material
#8. Culm margins <indumentum>/
  1. glabrous/
  2. scabrous/
  3. puberulous/
  4. pilose/
  • Assessed along total length
#9. Culm margin hairs <orientation>/
  1. antrorse/
  2. retrorse/
  3. divergent/
#10. Culm margin hairs <whether clumped>/
  1. clumped/
  2. evenly spaced/
#11. Culm margin hairs <whether embedded in resin>/
  1. embedded in resin/
  2. not embedded in resin/
#12. Culm margin hairs <position >/
  1. restricted to the apex of culm/
  2. along the upper third of culm/
  3. continuous along culm/
  4. along the lower third of culm/
  5. along mid-third of culm/
#13. Culms <length relative to leaves>/
  1. longer than leaves/
  2. more or less equal to leaves/
  3. shorter than leaves/
  • Culm length includes inflorescence (see character 2)
#14. Culm bases <colour>/
  1. green/
  2. yellow-green/
  3. pale yellow/
  4. pale yellow-brown <straw-coloured>/
  5. pale brown <pallid>/
  6. orange-brown/
  7. mid brown <tan>/
  8. dark brown <dark chocolate>/
  9. black-brown <intense dark brown>/
  10. pink-brown/
  11. red-brown/
  12. red-pink/
  13. dark red/
  14. purplish/
  15. black/
#15. Culm to leaf ratio <of lengths>/
  • Relative lengths of longest culm and leaf
#16. Leaves <whether equitant>/
  1. equitant/
  2. not equitant/
#17. Leaves <angle of fan (ramet) in degrees; Barrett 2007a>/
  degrees/
  • Measured as the angle between the two outermost leaves on a single complete ramet/fan
#18. Leaves <longest leaf; length>/
  mm long/
#19. Leaf sheaths <length>/
  mm long/
#20. Leaf sheaths <colour>/
  1. green/
  2. yellow-green/
  3. pale yellow/
  4. pale yellow-brown <straw-coloured>/
  5. pale brown <pallid>/
  6. orange-brown/
  7. mid brown <tan>/
  8. dark brown <dark chocolate>/
  9. black-brown <intense dark chocolate>/
  10. pink-brown/
  11. red-brown/
  12. red-pink/
  13. dark red/
  14. purplish/
  15. black/
  • Assessed under dissecting microscope
#21. Leaf sheaths <whether resinous>/
  1. resinous/
  2. not resinous/
  • Unopened sheath observed; comment on quantity
#22. Leaf sheath margins <opacity>/
  1. translucent/
  2. opaque/
#23. Leaf sheath margins <indumentum>/
  1. glabrous/
  2. scabrous/
  3. puberulous/
  4. pilose/
  • Along margin towards apex
#24. Leaf sheath face <indumentum>/
  1. glabrous/
  2. scabrous/
  3. puberulous/
  4. pilose/
#25. Ligule <apex shape>/
  1. caudate/
  2. accuminate/
  3. acute/
  4. obtuse/
  5. rounded/
  6. emarginate/
  7. lobed/
  8. truncate/
#26. Ligule <indumentum>/
  1. glabrous/
  2. scabrous/
  3. puberulous/
  4. pilose/
  • Refers to the apex of the ligule.
#27. Leaf lamina <whether well-developed and photosynthetic at maturity>/
  1. well-developed <photosynthetic, and at least some leaves shortly or distinctly laminate>/
  2. reduced <functionally elaminate; leaves with lamina much reduced or absent>/
#28. Leaf lamina <whether rigid or flaccid>/
  1. rigid/
  2. flaccid/
#29. Leaf lamina <longest leaf; length>/
  mm/
#30. Leaf lamina <width at mid-third of lamina length; longest leaf>/
  mm wide/
#31. Leaf lamina <thickness in mid-third; not equivalent to width>/
  mm thick/
#32. Leaf lamina <width to thickness ratio>/
  • Measured in mid-third of lamina length
#33. Leaf lamina <general shape in cross-section>/
  1. dorsiventrally flattened/
  2. terete/
  3. semiterete/
  4. isobilaterally oblong/
  5. isobilaterally flattened/
  6. isobilaterally fusiform/
#34. Leaf lamina <colour of margins>/
  1. with red margins/
  2. with colourless margins/
  3. with concolorous margins <i.e., margins same colour as faces>/
  4. with brown margins/
  5. with golden-orange margins/
#35. Leaf lamina <when dried; whether finely striate>/
  1. striate/
  2. not striate/
#36. Leaf lamina <whether deeply grooved>/
  1. deeply grooved/
  2. not deeply grooved/
#37. Leaf lamina margins <indumentum>/
  1. glabrous/
  2. scabrous/
  3. papillose/
  4. puberulous/
  5. pilose/
  • Assessed along whole length of lamina
#38. Leaf lamina margin hairs <orientation>/
  1. antrorse/
  2. retrorse/
  3. divergent/
#39. Leaf lamina marginal hairs <whether clumped>/
  1. clumped/
  2. evenly spaced/
#40. Leaf lamina marginal hairs <whether embedded in resin>/
  1. embedded in resin/
  2. not embedded in resin/
#41. Leaf lamina marginal hairs <position >/
  1. restricted to the lamina apex/
  2. along the upper third of lamina/
  3. continuous along lamina/
  4. along the lower third of lamina/
  5. along the lower half of lamina/
  6. along mid-third of lamina/
  7. along upper half of lamina/
#42. Leaf lamina to sheaths ratio <of lengths>/

**Inflorescence morphology:**

#43. Inflorescence <outline; i.e. overall plane shape>/
  1. broadly ovate/
  2. ovate/
  3. narrowly ovate/
  4. linear/
  5. oblong/
  6. narrowly oblong/
  7. circular/
  8. elliptic/
  9. narrowly elliptic/
  10. obovate/
  11. narrowly obovate/
  12. oblate/
  13. fan-shaped/
  14. narrowly rhombic/
  15. broadly rhombic/
#44. Inflorescence <form>/
  1. single spikelet/
  2. panicle-like/
#45. Inflorescence <length>/
  mm long/
  • Measured from the base of the lowest primary inflorescence bract to tip of distal spikelet.
#46. Inflorescence <width at widest point>/
  mm wide/
#47. Inflorescence <number of nodes on primary axis>/
  noded/
  • Score the maximum number per specimen; start with lowest node
#48. Inflorescence lowest internode <length along main axis>/
  mm long/
  • Measured from the base of lowest primary inflorescence bract to the base of the next sheath on the main inflorescence axis.
#49. Inflorescence lateral branches <number per node>/
  per node/
  • Scored for first lateral branch only
#50. Inflorescence lateral branches <whether elongated>/
  1. elongated <branch visible above sheath of inflorescence bract>/
  2. contracted <not visible beyond sheath>/
  • Scored only where lateral branches with more than one spikelet were present.
#51. Inflorescence lateral branches <longest branch; length>/
  mm long/
  • Measured from lowest node to tip of the distal spikelet
#52. Proximal <inflorescence> lateral branch <length>/
  mm long/
#53. Proximal <inflorescence> lateral branch <width>/
  mm wide/
#54. Involucral bract <total length>/
  mm/
#55. Involucral bract to inflorescence ratio <of total lengths>/
#56. Involucral bract sheath <length>/
  mm/
#57. Involucral bract sheath <colour>/
  1. green/
  2. yellow-green/
  3. pale yellow/
  4. pale yellow-brown
  5. pale brown/
  6. orange-brown/
  7. mid brown/
  8. dark brown/
  9. black-brown/
  10. pink-brown/
  11. red-brown/
  12. red-pink/
  13. dark red/
  14. purplish/
  15. black/
  16. grey/
#58. Involucral bract sheath <indumentum>/
  1. glabrous/
  2. scabrous/
  3. puberulous/
  4. pilose/
#59. Involucral bract sheath apex <indumentum>/
  1. glabrous/
  2. scabrous/
  3. puberulous/
  4. pilose/
#60. Involucral bract sheath margin <opacity>/
  1. translucent/
  2. opaque/
#61. Involucral bract sheath ligule <whether with a free limb>/
  1. with a free limb/
  2. without a free limb/
#62. Involucral bract sheath ligule <apex shape>/
  1. caudate/
  2. acuminate/
  3. acute/
  4. obtuse/
  5. emarginate/
  6. lobed/
  7. rounded/
  8. truncate/
#63. Involucral bract sheath ligule <indumentum>/
  1. glabrous/
  2. scabrous/
  3. puberulous/
  4. pilose/
#64. Involucral bract lamina to sheath ratio <of lengths>/
#65. Involucral bract <whether with a well-developed lamina>/
  1. lamina well-developed <photosynthetic>/
  2. reduced <non-photosynthetic at maturity>/
  • Lamina well-developd or much narrower than sheath or mucro-like or absent
#66. Involucral bract lamina <length>/
  mm long/
#67. Involucral bract lamina <width>/
  mm wide/
#68. Involucral bract lamina <shape in cross-section>/
  1. isobilaterally flattened <or elliptical> along entire length <of lamina>/
  2. triangular at lamina apex <but becoming isobilaterally elliptical above ligule; e.g. *Lepidosperma tortuosum*>/
  3. triangular along entire length <of lamina>/
  4. triangular at lamina apex <and becoming half-rhombic towards base>/
  5. triangular at lamina apex <and becoming trullate towards base>/
  6. triangular at lamina apex <and becoming circular towards base>/
  7. circular along entire length/
  8. dorsiventrally flattened along entire length/
  9. dorsiventrally flattened at apex <and becoming oblong at base>/
  10. dorsiventrally crescentiform at apex <and becoming isobilaterally elliptical towards base>/
#69. Involucral bract lamina <indumentum>/
  1. glabrous/
  2. scabrous/
  3. puberulous/
  4. pilose/
#70. Involucral bract lamina apex <shape>/
  1. aristate/
  2. caudate/
  3. subulate/
  4. acuminate/
  5. acute/
  6. obtuse/
  7. rounded/
#71. Rachis <whether flexuous>/
  1. flexuous/
  2. not flexous/
  • Spikelet-bearing axis of inflorescence.
#72. Rachis <whether reflexed>/
  1. reflexed <e.g. *Lepidosperma tortuosum*>/
  2. not reflexed/

**Spikelet and floral morphology:**

#73. Spikelet prophyll <shape of apex>/
  1. obtuse/
  2. truncate/
  3. emarginate/
  4. acute/
#74. Spikelet prophyll margin <indumentum>/
  1. glabrous/
  2. scabrous/
  3. puberulous/
  4. pilose/
#75. Spikelet prophylls outside <abaxial> face <indumentum>/
  1. glabrous/
  2. scabrous/
  3. puberulous/
  4. pilose/
#76. Spikelet prophyll keel <indumentum>/
  1. glabrous/
  2. scabrous/
  3. puberulous/
  4. pilose/
#77. Spikelets <number per inflorescence>/
  1. less than 30/
  2. more than 30/
#78. Spikelets <number per lowest lateral branch>/
  per lowest lateral branch/
#79. Spikelets <length excluding any pedicel>/
  mm long/
  • Measure spikelet from base to apex
#80. Spikelets <diameter>/
  mm diameter/
#81. Sterile floral bract apex <shape>/
  1. aristate/
  2. subulate/
  3. acuminate/
  4. acute/
  5. obtuse/
  6. rounded/
  7. truncate/
  8. emarginate/
#82. Sterile floral bract midrib <whether raised>/
  1. raised/
  2. flush/
#83. Sterile floral bract <whether mucronate>/
  1. mucronate/
  2. muticous <not mucronate>/
#84. Sterile floral bract outside (abaxial) face <indumentum>/
  1. glabrous/
  2. scabrous/
  3. puberulous/
  4. pilose/
#85. Sterile floral bract margins <form of margin indumentum>/
  1. glabrous/
  2. scabrous/
  3. puberulose/
  4. pilose/
#86. Sterile floral bracts <number per spikelet>/
  per spikelet/
#87. Fertile floral bracts <number per spikelet>/
  per spikelet/
#88. Fertile female floral bract apex <shape>/
  1. aristate/
  2. subulate/
  3. acuminate/
  4. acute/
  5. obtuse/
  6. rounded/
#89. Fertile floral bracts <whether mucronate>/
  1. mucronate/
  2. muticous <not mucronate>/
#90. Fertile floral bract midrib <whether obvious at 10x>/
  1. apparent/
  2. indistinct/
#91. Fertile floral bract midrib <indumentum>/
  1. glabrous/
  2. scabrous/
  3. puberulous/
  4. pilose/
#92. Fertile floral bracts outside face <indumentum; abaxially, excluding midrib and margins>/
  1. glabrous/
  2. scabrous/
  3. papillose/
  4. puberulous/
  5. pilose/
#93. Fertile floral bracts inside face <indumentum; adaxially, excluding margins>/
  1. glabrous/
  2. scabrous/
  3. puberulous/
  4. pilose/
#94. Fertile floral bracts margins <form of margin indumentum>/
  1. glabrous/
  2. scabrous/
  3. puberulous/
  4. pilose/
#95. Fertile floral bract nerves <number visible with 10x magnification>/
#96. Female-fertile flowers <includes bisexual flowers; number>/
  per spikelet/
#97. Male flowers <number>/
  per spikelet/
#98. Female-fertile floral bracts <length>/
  mm long/
#99. Female-fertile floral bracts <width; measure *d in si* tu>/
  mm wide/
#100. Total number of flowers per spikelet/
#101. Stamens <number per flower>/
  per flower/
#102. Anther <length excluding apiculus>/
  mm long excl. apiculus/
#103. Anthers <width>/
  mm wide/
#104. Anther apiculus <length>/
  mm long/
#105. Anther apiculus <indumentum>/
  1. glabrous/
  2. scabrous/
#106. Style branches <number>/
  per pistil/

**Fruit and perianth morphology:**

#107. Perianth scale <number>/
#108. Perianth scale <shape>/
  1. narrow ovate/
  2. ovate/
  3. broadly ovate/
  4. circular/
#109. Perianth scale apex <shape>/
  1. caudate <drawn into long tail>/
  2. subulate/
  3. acuminate/
  4. acute/
  5. rounded/
  6. obtuse/
#110. Perianth scale apex <indumentum>/
  1. glabrous/
  2. indumented/
#111. Perianth scale apex hairs <position>/
  1. restricted to the very tip/
  2. not restricted to the tip <and extending proximally>/
#112. Perianth scale fused base <maximum length>/
  mm/
#113. Perianth scale free limb <maximum length>/
  mm/
#114. Perianth scale <width>/
  mm/
  • Measured at widest part of scale
#115. Perianth scales <whether overlapping at the base >/
  1. overlapping/
  2. not overlapping/
#116. Fruit <length including perianth>/
  mm long including perianth/
#117. Fruit <length excluding perianth>/
  mm long excluding perianth/
#118. Fruit <diameter>/
  mm diameter/
  • At widest diameter
#119. Fruit <shape in broadest lateral view>/
  1. spherical/
  2. ovate <widest near base>/
  3. oblong <with parallel sides>/
  4. elliptical <widest in middle >/
  5. obovate <widest near apex>
#120. Fruit ribs <whether distinct from fruit faces>/
  1. discolorous with faces/
  2. concolorous with faces/
#121. Fruit ribs <whether raised>/
  1. raised <above face of fruit>/
  2. not raised <ribs more or less flush with surface of nut>/
  3. sunken <below face of fruit>/
#122. Fruit ribs <colour>/
  1. green/
  2. pale green/
  3. pale yellow/
  4. yellow-brown/
  5. pale brown/
  6. orange-brown/
  7. mid brown/
  8. dark brown/
  9. red-brown/
  10. black/
#123. Fruit face <colour at maturity>/
  1. green/
  2. pale green/
  3. pale brown-green/
  4. yellow-green/
  5. pale yellow/
  6. yellow-brown/
  7. pale brown/
  8. orange-brown/
  9. mid brown/
  10. dark brown/
  11. red-brown/
  12. black/
#124. Fruit faces <whether wrinkled at maturity>/
  1. wrinkled/
  2. not wrinkled/
#125. Fruit style cap <length>/
  mm/
#126. Fruit style cap apex <shape>/
  1. truncate/
  2. rounded/
  3. obtuse/
  4. acute/
#127. Fruit style cap <colour>/
  1. green/
  2. pale green/
  3. pale brown-green/
  4. yellow-green/
  5. pale yellow/
  6. yellow-brown/
  7. pale brown/
  8. orange-brown/
  9. mid brown/
  10. dark brown/
  11. red-brown/
  12. black/
  • Record if fruit immature, recently mature or well mature.
#128. Fruit style cap <indumentum>/
  1. glabrous/
  2. scabrous/
  3. papillous/
  4. <minutely> colliculate/
#129. Fruit style base <presence fused on top of style cap>/
  1. present/
  2. absent/
#130. Fruit style base <length>/
  mm/
#131. Fruit style base <width>/
  mm/

**Culm anatomy**

• As seen in cross-section. Many characters are from or modified from Metcalfe (1971) with others from Bruhl (1995) and our subsequent work. Cross-sections made from mid-third of culm.

#132. Culms <shape in cross-section>/
  1. triangular/
  2. circular/
  3. truncated circular/
  4. subhemispherical/
  5. square/
  6. trullate or rhombic/
  7. crescentiform/
  8. fusiform/
  9. broadly elliptical/
  10. elliptical/
  11. narrowly elliptical/
  12. ovate/
  13. ‘almond-shaped’<ovate with one margin acute and the other obtuse or rounded>/
  14. oblong/
  15. shallowly hexagonal/
  16. ‘Swiss-escutcheon-shaped’<shaped like a Swiss escutcheon, which is an elaborated heart shape with two convex sides and a truncate apex with a short projection in the middle >/
#133. Culm margins <shape in cross-section>/
  1. rounded <e.g. in *Lepidosperma viscidum*>/
  2. obtuse/
  3. acute/
  4. acuminate/
#134. Culm stomata <position relative to epidermal cells>/
  1. raised/
  2. flush/
  3. sunken/
#135. Culm vascular bundles <number>/
#136. Culm vascular bundles <number of distinct size classes>/
#137. Culm vascular bundles enclosed within chlorenchyma <presence>/
  1. present/
  2. absent/
  • Individual vascular bundles surrounded by chlorenchyma cells
#138. Culm vascular bundles enclosed within parenchyma <presence>/
  1. present/
  2. absent/
  • Individual vascular bundles surrounded by parenchyma cells
#139. Culm vascular bundles between chlorenchyma and parenchyma <presence>/
  1. present/
  2. absent/
  • Individual vascular bundles may be positioned between, and contacting both, chlorenchyma and parenchyma, i.e. not completely surrounded by one tissue type.
#140. Culm sclerenchymatous fibre caps <presence>/
  1. present/
  2. absent/
#141. Culm sclerenchymatous fibre girders <presence>/
  1. present/
  2. absent/
  • Girders connecting vascular bundles and epidermis.
#142. Culm sclerenchymatous fibre girders between vascular bundles <presence>/
  1. present <e.g. in *Lepidosperma concavum* (*J.J. Bruhl 25* 74)>/
  2. absent/
#143. Culm sclerenchymatous fibre strands <presence>/
  1. present/
  2. absent/
#144. Culm sclerenchymatous fibre strands <whether all contacting epidermis>/
  1. all contacting epidermis/
  2. some not contacting epidermis/
  • Some small strands occur which are surrounded by mesophyll or parenchyma and do not contact the epidermis or vascular bundles.
#145. Culm pith or parenchyma <whether homogeneous or heterogeneous>/
  1. homogeneous/
  2. heterogeneous/
  • Homogeneous culm pith /parenchyma consists mainly of a single, uniform tissue type. Heterogeneous pith is composed of two distinct tissue types: (1) tissue where the cells have relatively thick cell-walls, usually associated with the vascular bundles; and (2) ‘translucent tissue’ where the cells are generally larger and have thin cell-walls.
#146. Culm pith or parenchyma <heterogeneous morphology>/
  1. vascular bundle ‘rings’ surrounding translucent tissue/
  2. large amount of pith with thin ‘bridges’ between opposite vascular bundles/
#147. Culm multicellular trichomes <whether present>/
  1. absent/
  2. present singularly/
  3. present in groups/
#148. Culm epidermal zones <relative width>/
  1. wider costally/
  2. equal in width/
  3. wider intercostally/
#149. Culm epidermal cell silica bodies <presence>/
  1. present <within thin-walled epidermal cells>/
  2. absent <and epidermal cells thick walled>/
#150. Culm epidermal cell silica bodies <distribution>/
  1. throughout epidermis/
  2. restricted to margin epidermis/
#151. Culm epidermal ‘silica cells’ <position relative to non-silica epidermal cells>/
  1. raised/
  2. flush/
  3. sunken/
  • ‘Silica cells’ are epidermal cells containing one or more silica bodies
#152. Culm sub-epidermal sclerenchymatous strands and/or girders width <mean number of cells>/
  • Mean of counts of five adjacent strands and/or girders. Within each strand and/or girder count the number of sclerenchymatous cells adjacent to the epidermis as seen in cross-section. Counted in mid-third of section if possible.
#153. Culm sub-epidermal sclerenchymatous strands and/or girders length <mean number of cells>/
  • Mean count of five adjacent strands and/or girders. Within each strand and/or girder count the number of sclerenchymatous cells from its inner end to the epidermis, not including the mestome sheath. Counted in mid-third of section where possible.

## Appendix 2

**Table:** Dataset of *Lepidosperma* species from DELTA file. **Operational taxonomic unit** (OTU) code in first column. Characters marked with * not included in phenetic analysis. See Table 1 for OTU details and Appendix 1 for character list.

Character	curBag01	curCon01	curMac01	curRos01	eldCou01	eldEld01	eldFie01	eldFie02	eldMcC01	eldPro01	eldSpr01	eldSpr02	eldSpr03	inoBre01	inoGra01	inoLen01	inoUni01	inoUni02	torBaro02	torBarT01	torBla01	torBoo01	torDem01	torMul01	torWer01
**1***	2	1/2	2	2	2	2	2	2	2	2	2	2	2	2	2	2	2	2	2	2	2	2	2	2	2
**2**	99	116	93	185	31	43	51	35	48	41	51	55	39	49	17.5	45	45	56	341	233	423	350	319	343	502
**3**	1.23	1.34	1.21	1.21	1.18	1.23	0.8	0.81	0.97	1.3	1.4	1.24	1.31	0.74	0.83	1.02	0.72	0.67	1.44	0.83	0.82	1.55	1.21	1.07	0.82
**4**	0.39	0.41	0.72	0.65	0.36	0.44	0.36	0.39	0.46	0.27	0.51	0.28	0.39	0.42	0.47	0.41	0.35	0.38	0.62	0.61	0.6	0.77	0.55	0.45	0.66
**5**	3.15	3.27	1.68	1.86	3.28	2.8	2.22	2.08	2.11	4.81	2.75	4.43	3.36	1.76	1.77	2.49	2.06	1.76	2.32	1.36	1.37	2.01	2.2	2.38	1.24
**6***	2	1	2	1/2	1	1	1	1	1	1	1	1	1/2	2	2	2	1	1	1	1	1	1	1	1	1
**7***	2	2	2	2	2	2	2	2	2	2	2	2	2	2	2	2	2	2	1/2	2	1/2	1/2	1/2	2	2
**8**	2	2	2	2	2	1&2	1&2	1/2	2	2	1/2	1/2	1/2	1	1	1/2	1/2	1/2	2	2	2	2	2	2	1/2
**9**	3	3	3	3	1	1	1	1	1	1	1	1	1			1	1	1	3	3	1/3	1	3	3	3
**10**	2		2	2	2	2	1	1/2	1/2	1/2	2	1/2	2			2	1	2	2	1/2	1	1	2	2	1/2
**11***	2	2	2	2	2	2	2	2	2	2	2	2	2			2	2	2	2	2	2	2	2	2	2
**12**	3	3	3	2& 3/5	3	3	2/4	1/3	3	3	3	2/3	2/3			2	1	2	3	3	3	3	3	3	3
**13**	3	3	3	3	3	3	3	3	3	3	3	3	3	3	3	3	3	3	1	2	1	1	1	1	1
**14***	3/5	3	3/5	3/5	2/3	3/4	3/5	3	2/4	1/3-4	3	3	3	3	3	3/5-7	3	3	3	7-11-13	11/12	3	2/3/5	3/5	3/12
**15**	0.36	0.47	0.54	0.82	0.56	0.59	0.58	0.7	0.66	0.53	0.57	0.69	0.6	0.35	0.27	0.34	0.37	0.31	1.45	1.16	3.38	1.66	1.29	1.66	1.56
**16***	1	1	1	1	1	1	1	1	1	1	1	1	1	1	1	1	1	1	1	1	1	1	1	1	1
**17**	10	10	15	6	22	20	20	20	23	24	26	27	25	12	15	15	15	9	7	5	5	8	5	6	7
**18**	272	246	173	225	55	73	88	50	73	77	79	80	65	139	65	133	121	181	235	227	125	211	247	207	321
**19**	40	34	25	26	16	22	25	12	14	23.5	22	22	12	29	15	30	19.5	36.5	35	26	50	25	23	20	31
**20**	3/5	4/7	3/5	4/5	3/4-5/12	3/12	1/3-4/5-12	3/5-12	3/4-5/7-12	5/7-12	3/5-7/12	3/5-7/12	2/3-4/5-7/11	3/5	4/5-7	4/5-7	3/5-7	1/5-7	3/4-5/7	2/4-5/7-12	8/11<br/> /12	3/4-5	3/4	3/4-5	3/7-11/12
**21**	1	1	1	1	2	2	2	2	2	2	2	2	2	2	1	1/2	2	2	2	2	2	2	2	2	2
**22***	1/2	1	1	1	1	1	1	1	1	1	1	1	1	1	1	1	1	1	1	1	1	1	1	1	1
**23***	1	1	1	1	1	1	1	1	1	1	1	1	1	1	1	1	1	1	1	1	1	1	1	1	1
**24***		1			1	1	1	1	1	1	1	1	1				1	1							
**25**	4/6	4/5	4/6	3/4	2/3	1/2	2	3	2	1/2	2/3	2	2	2	2/3	2/3	1	1	6	6	6	4/6	8	6	6
**26***	1	1	1	1	1	1	1	1	1	1	1	1	1	1	1	1	1	1	1	1	1	1	1	1	1
**27***	1	1	1	1	1	1	1	1	1	1	1	1	1	1	1	1	1	1	1	1	1	1	1	1	1
**28***	1	1	1	1	1	1	1	1	1	1	1	1	1	1	1	1	1	1	1	1	1	1	1	1	1
**29**	232	212	148	199	39	51	63	38	59	63.5	68	58	53	110	50	103	101.5	144.5	200	201	75	86	224	187	279
**30**	1.4	1.43	1.76	1.18	1.15	1.24	1.15	0.99	1.01	1.31	1.55	1.44	1.46	0.71	1.06	0.95	0.88	1.2	1	0.63	0.63	1.32	1.39	1.22	0.77
**31**	0.4	0.4	0.52	0.49	0.32	0.32	0.31	0.35	0.21	0.29	0.48	0.29	0.3	0.19	0.22	0.34	0.18	0.22	0.33	0.36	0.39	0.44	0.34	0.31	0.5
**32**	3.5	3.57	3.38	2.41	3.59	3.87	3.71	2.83	4.81	4.52	3.23	4.97	4.87	3.74	4.82	2.79	4.89	5.45	3.03	1.75	1.62	3	4.09	3.94	1.54
**33***	5	5	5	5	5	5	5	5	5	5	5	5	5	5	5	5	5	5	5	5	5	5	5	5	5
**34**	2/3	2/3	3	3	2/3	3	2/3	3	3	2/3	2/3	2/3	2/3	3	3	3	3	3	2/3	2/3	3	2	2/3	2/3	3
**35***	2	1	2	1/2	1	1	1	1	1	1	1/2	1	1	1	1	1/2	1	1	1	1/2	1	1	1	1	1
**36**	2	2	2	2	2	2	2	2	2	2	2	2	2	2	2	2	2	2	2	1	1/2	1	1	1	1/2
**37**	2	2	2	2	1/2	2	1/2	1/2	1/2	1/2	1/2	1/2	1/2	1/2	1/2	1/2-4	1/2	2	1/2	1/2	2	2	2	2	1/2
**38**	1/3	3	1/3	3	1	1	1	1	1	1	1	1	1	1	1	1	1	1	3	3	1	1/3	3	1/3	1
**39**	2	2	1	2	1	1/2	1	1	1/2		1/2	1/2	2	2	1/2	1/2	1/2	1	2	1/2	1	1	2	1/2	1
**40***	2	2	2	2	2	2	2	2	2	2	2	2	2	2	2	2		2	2	2	2	2	2	2	2
**41**	3	3	3	3	1/2	2/3	2/3	1/2	1/2	1	1/2/6	1/6	2/3	7	7	7	1		3	3	1	3	3	3	3
**42**	5.8	6.24	5.92	7.65	2.44	2.32	2.52	3.17	4.21	2.28	3.09	2.64	4.42	3.79	3.33	3.43	5.21	3.96	5.71	7.73	1.5	3.44	9.74	9.35	6.64
**43**	9	2/3/<br/> 8/9	6/9	2/8	10	10	2	2	10	10	1/2	2/10	1/2	3	3	3	3	3	2/5	1/8	2/5/8	1/5	2/8	2	1/12
**44**	2	2	2	2	2	2	1/2	1	1/2	1/2	1/2	1/2	1/2	2	1/2	1/2	1/2	2	2	2	2	2	2	2	2
**45**	30	29	30	35	7	9.5	12	9	10	10.5	8.5	8.5	9	10	5.5	12	11	7.7	12	9	12	11	7.5	11	11
**46**	10	7	8	13	3.5	2.5	5	3	10	5	2.5	4	5.5	3.5	4	7	3.5	4.4	12	12	9	8	10	11.5	10.5
**47**	7	8	7	7	2	3	2	1	3	3	2	2	2	2	2	2/3	2	3	3	3	4	4	4	3	5
**48**	8	8	10.5	9.5	1		3.5		2.5	2	2.5	2	2.5	4.5	2	6.5	5.5	2	4	2	7	4	2.5	3	3
**49***	1	1	1	1	1	1	1		1	1	1	1	1	1	1	1	1	1	1	1	1	1	1	1	1
**50**	1/2	1/2	1/2	1/2															2		1	2	2		2
**51**	16	16	15	22	6.5	5	7		8	8.5	7.5	5	6.5	6	5	7.5	6	6.1	12.5	9	12	11	9.5	11	11
**52**	16	16	15	22	6.5	5	7		8	8.5	7.5	5	6.5	6	5	7.5	6	6.1	12.5	9	12	11	9.5	11	11
**53**	4	2.5	3	5	1.5	1	2		2	1.5	1	1.5	2	1.5	2	4	2	1.5	7.5	3	7	8	5	2.5	7
**54**	19	2.5	21.5	22	9	12	20.5	13	10.5	24.5	12	9.5	10.5	52	27	59	49	41	13	9.5	28	11	8	9.5	10
**55**	0.63	31	0.72	0.63	1.29	1.26	1.71	1.44	1.05	2.33	1.41	1.12	1.17	5.2	4.91	4.92	4.45	5.32	1.08	1.06	2.33	1	1.07	0.86	0.91
**56**	10	1.07	10.5	10	5	5.5	7.5	7	5.5	8	7	5.5	5.5	10	6.5	9	7.5	5.5	3	2	4	4	2	2.5	2.5
**57**	5/7-16	5	5/7	5/7-8	7/11<br/> /13	5/7-11	5/7-8	5/7-8/9-11	7/8-11/13	5/7-8/12	5/7	7/8	7/8	2	1/3-4	3/5-7	3/4-5	1/3-5/7	4/7-11	7/11	11/13	3/7-8/12	7/8	7/8	8/11
**58**	2	2	2	2/3	2	2	1	1	2	1/2	1	1	1	2	1	2/3	2	2	1	2	1	3	2	2	1
**59**	2	2	1/2	1/2	1	1	1	1	1	1	1	1	1	1	1	1	2	2	1	1	1	1	1	1	1
**60**	2	1	1	2	1	1	1	1	1	1/2	1	1	1	1	1	1	1	1	1	1	1	1	2	1	1
**61***	1	1	1	1	1		1	1		1	1	1	1	1	1	1	1	1	1	1	1	1	1	1	1
**62**	3/4	4	5	2/3	1	1	1	2		1	1	1	2	2	2	2	1	1	5	5	5	5	5	5/8	5
**63**		2	2		1	1	1	1		1	1	1	1	1	1	1	1	1	1	1	1	1	1	1	1
**64**	0.9	2.44	1.05	1.2	0.8	1.18	0.52	0.57	0.91	2.06	1.4	0.73	0.91	4.2	3.15	5.56	5.53	6.45	3.33	3.75	6	2.25	3	0.74	3
**65***	1	1	1	1	1	1	1	1	1	1	1	1	1	1	1	1	1	1	1	1	1	1	1	1	1
**66**	9	22	11	10	4	6.5	13	6	5	16.5	5	4	5	42	20.5	50	41.5	35.5	10	7.2	24	9	6	7	7.5
**67**	0.88	1.04	0.75	1.1	0.72	0.65	0.89	0.56	0.61	1.24	0.43	0.74	0.74	0.85	1	1.11	0.65	0.82	0.97	0.72	0.73	0.95	0.68	0.51	0.51
**68**	2/3	2/3	2/3	2/3	2/10	10	10	2	10	2/10	2/10	2	2/10	2	2	2	2	2	2	1/2/3	2	2	2	2/3	2
**69**	2	2	2	2	2	2	2	2	2	2	2	2	2	2	1/2	2/3	2	2	1/2	2	2	2/3	2	2	1/2
**70**	4	2/4	4		5	5/7	2	7	5/6/7	4	7	7	7	4	4	4	4	4	4	4	3/4	3/4	4	4	5/3
**71***	2	2	2	2	2	2	2		2	2				2	2	2	2	2	2	1	1	1	2	1/2	1/2
**72**	2	2	2	2	2	2	2		2	2	2	2	2	2	2	2	2	2	1	1	1	1	1	1	1
**73**	3	4	3	3	4	3	4		3/4	3/4				3/4	2/4	4	4	4	3	1		3	3	3/4	3/4
**74**	3	3	3	3	3	3	1		3	1				3	2	1	1	3	1	1	3	3	1	1	1
**75**	1	1	1	1	1/2	3	1		1	1				1	1	1	1	1	1	1		1	1	1	1
**76**	3	3	3	3	3/4	3	3		3/4	3				3	3	3	3	3	2	3		3/4	2/3	3	3
**77***	1	1	1	1	1	1	1	1	1	1				1	1	1	1	1	1	1	1	1	1	1	1
**78**	4	5	4	6	1	1	1	1	1	1		1		1	1/2	1	1	1	3	1	3	3	2	1	4
**79**	6.5	6	3.6	6.3	6.5	5	6.5	6.5	6.5	6.5		5		5.4	4.8	4.5	5.3	5.6	7.8	6	5.3	6.8	6.8	6.1	6.7
**80**	1.6	1.2	1.6	1.7	1.5	1	2	2	1.6	1.7		1.5		1.3	2.3	2	2	1.5	2.6	1.5	1.2	1.9	1.6	1.35	1.4
**81***	1		1	1						1				2	1/2	1	1		8		7/8	7	1	8	8
**82***	1		1	1						1				2	1	1	1		2		1	1	1	1	1
**83***	1		1	1						1				2	2		1		1		1	1	1	1	1
**84***	2		3	2						2				2	1	1	1		1		2	2	1	1/2	1/2
**85***	3		3	3						1				2	3	1	2		1		3	3	1	1	1/2
**86***	2		3	3						2				3	3	3	3		1		3		2	3	5
**87***	1		3	2						1				1	1	2	2		1		1		2	2	3
**88***	3		2	2						2				2/3	2	2	3		2		4	3	2	3	2
**89***	2		2	2						2				2	2	2	2		2		1	1	2	2	2
**90***	1		1	1						2				1	1	1	2		1		1	1	1	1	2
**91***	2		2	3						3				2	2	3	1		2		3	3	1/2	3	2
**92***	2		2	4						4				2	2	4	2		2		4	4	1/2	4	2
**93***	1		2	3						1				1	1	1	1		1		1	1	1	1	1
**94***	3		3	3						1				3	3	3	2		1/2		3	3	1	3	2
**95***	1		1	2						0				1	2	3	0		1		1	1	1	2	0
**96***	1		3	2						1				1	1	2	2		1		1		1	1	1
**97***	1		3	2						1				1	1	2	2		2		1		2	2	3
**98***	3.7		3.4	5.4						5.7				3.7	4.8	3.1	2.5		6		4.4		5	4.4	5.05
**99***	0.9		0.6	0.5						1				0.8	1.25	0.9	4		1.1		0.9		0.9	0.6	0.8
**100***	1		3	2						1				1	2	2	2		2		1		2	2	3
**101***			3	3						3				3		3	3		3				3	3	3
**102***			0.9	1.6						1.4				1.5	1.15				1.7		1.8				
**103***			0.35	0.3						3.5				0.2	0.25				0.3		0.2				
**104***			0.3	0.5						0.4				0.45	0.2				0.2		0.2				
**105***			2	1						1				2	1						1/2				
**106***			3	3			3							3	3	3								3	3
**107**	6/7	6	6	7		6	8	6	6		8	6	6		6				6	6		6	6		6
**108***	1	1	1	1		1	1	1/2	1		1	1	1		1/2				1	1/2		2	1		1/2
**109***	3/4	2	2/3	3		3/4	2/3	3	3		3	3	2/3		2/3				3/4	3		3/4-5	3		2/3
**110***	1/2	1	1/2	1		1	1	1	1		1	1	1		1					1		1			
**111***	1		1																						
**112**	0.4	0.2	0.05	0.3		0.1	0.4	0.3	0.3		0.1	0.3	0.1		0.2				0.2	0.2		0.1	0.35		0.4
**113**	1.1	0.8	1.05	1.3		1.1	1.6	0.8	1.5		1.1	1.5	0.9		0.8				0.9	0.8		0.9	0.6		1.2
**114**	0.4	0.5	0.5	0.4		0.4	0.5	0.6	0.5		0.6	0.4	0.8		0.5				0.35	0.5		5.5	0.3		0.5
**115***	1	2	1	1/2		1/2	1	1/2	2		1	2	1		2				2	1		1	2		
**116**	3.6	3	2.65	3.1		3	3.4	3.1	2.95		3.05	3.1	3.15		2.7				2.8	2.5		2.8	2.6		2.8
**117**	3.2	2.8	2.6	2.8		2.9	3	2.8	2.65		2.95	2.8	3.05		2.5				2.6	2.3		2.7	2		2.6
**118**	1.5	1.6	1.35	1.7		1.65	1.7	1.6	1.4		1.5	1.45	1.7		1.4				1.45	1.5		1.5	1.4		1.45
**119***	4	4	4	5		4	4	4	4		4	4/5	4		4				2/4	4		4	4		4
**120**	1	1	1	1		1	2	1	1		1	2	2		1				1	1		1	1		1
**121**	1	1/2	1	1		1	2	2	1		2	1	2		1				1	1		1	1		1
**122***	3/5	3/5	5	5		1/3	5	3/5	2		6	2	7/8		3				3	3/5		3	3/5		3/4
**123***	1/3	2/7-9	9	7/9		3/9	9	9/10	4		3	2/7	10		7				1/7	3		3	1		3/9
**124***	2	2	2	2		2	2	2	2		2	2	2		1				1	2		2	2		1/2
**125**	0.3	0.2	0.3	0.4		0.3	0.25	0.6	0.2		0.2	0.3	3		0.4				0.4	0.3		0.3	0.3		0.4
**126***	1	1	2	1		1	1	1	1		1	1	1						1/2	3		1/3			1
**127***	10	5/9	7	7		5/10	8	10/12	2/5		10/12	7/9	12						11	7		5			6
**128**	1	1	1			4	4	4	4	4	4	4	4		1				1	1		1	1		1
**129***	1	1	1			1	1	1	1		1	1	1		1				2	1		1	1		1
**130**	0.1	0.05	0.05			0.1	0.2	0.2	0.13		0.2	0.1	1.5		0.05					0.1		0.1	0.1		0.1
**131**	0.1	0.1	0.15			0.15	0.2	0.2	0.2		0.2	0.2	2		0.1					0.15		0.1	0.1		0.15
**132**	10/11	10	11	11	13	6	13	13	13	13	13	10	10/13	14	9/14	10	9/12	2/9	13	16	15	4	15	15	16
**133**	3/4	4	3/4	2/3	1&3	1&3	1/3	1/4	1&4	1&4	2/3	2/3	2/3	1		1			3/1	2&4	1&2	3&4	3	4	1/2
**134***	2	1/2	2	2	2	2	2	2	2	2	2	2	2	2	2		2	2	2	2	2	2	2	2	2
**135**	32	38	29	23	13	13	10	10	10	10	11	12	15	13	11	9	10	12	26	17	18	31	21	2	19
**136**	3	2	3	2	2	2	2	2	2	2	2	2	2	2	2	2	2	2	2	3	3	3	3	3	3
**137***	1	1	1	1	1	1	1	1	1	1	1	1	1	1	1	1	1	1	1	1	1	1	1	1	1
**138**	1	1	1	2	2	1	2	2	2	2	2	2	1	2	2	2	2	2	1	2	2	1	2		2
**139**	1	1	1	1	1	1	1		1	1	1	1	1	1	2	1	1	1	1	1	1	1	1	1	1
**140***	1	1	1	1	1	1	1	1	1	1	1	1	1	1	1	1	1	1	1	1	1	2	1	1	1
**141**	1	1	1	1	2	2	2	2	2	2	2	2	2	2	2	2	2	2	2	2	2	2	2	2	2
**142***	2	2	2	2	2	2	2		2	1	2	2	2	2	2	2	2	2	2	2	2	2	2	2	2
**143***	1	1	1	1	1	1	1	1	1	1	1	1	1	1	1	1	1	1	1	1	1	1	1	1	1
**144***	1	1	1	1	1	1	1	1	1	1	1	1	1	1	1	1	1	1	1	1	1	1	1	1	1
**145**	2	2	1	2	2	2	2		2	2	2	2	2	2	2	2	2	2	1	1	1	1	1	1	1
**146**	1	1		2	1	1	1		1	1	1	1	1				1	1							
**147**	1	1	1	1	1	1	1	1	1	1	1	1	1	1	1	1	1	1	1	1	1	2	1	1	1
**148**	1	1	2/3	1/2	3	2	1/2	2	3	3	2	2	2	1	1	1	1	1	1	1/2	1	2	2	1	2
**149**	1	1	1	1	1	1	1	1	1	1	1	1	1	1	2	2			1	1	1	1	1	1	1
**150***	1	1	1	1	1	1	1	1	1	1	1	1	1	1					1	1	1	1	1	1	1
**151**	2	2	2	2	2/3	2/3	2	2	3	2/3	2	2/3	2	2					2	2	2	2	2	2	2
**152**	8	7	7.2	7.8	7.8	5.8	6.6	9	5.6	6	7	7.2	5.4	8.2	6.2	6.8	17	8	7.8	5.8	8.2	7	7.4	5.8	8
**153**	12.8	14	10.8	12.6	6.2	4.8	3.4	3.2	5	4.4	4.6	4.6	5	8	12.2	5.4	3.8	6	7.2	5.6	9.8	9.2	8.8	7.6	9.6

## Appendix 3

Index to numbered collections of *Lepidosperma* seen by G.T. Plunkett

All specimens are in HO unless otherwise indicated.

**Allan, M.** *s.n.* 30 Sept. 1975 HO 135425, *s.n.* 19 Oct. 1979 HO 515594 (*Lepidosperma inops*).

**Beauglehole, A.C.** 25084 MEL (*Lepidosperma curtisiae*).

**Brown, M.J.** 1365 (*Lepidosperma monticola*).

**Bruhl, J.J.** 630 (*Lepidosperma monticola*); 2407 NE (*Lepidosperma inops*); 2453 NE (*Lepidosperma tortuosum*); 3005 NE (*Lepidosperma curtisiae*).

**Buchanan, A.M.** 3293, 3018a, 7276, 7629 (*Lepidosperma inops*); 9981 (*Lepidosperma monticola*); 10880 (*Lepidosperma inops*); 12360 (*Lepidosperma monticola*); 13324, 14710, 15288 (*Lepidosperma inops*).

**Coates, F.** *s.n.* 25 Jan. 1990 HO 127069 (*Lepidosperma inops*).

**Collier, P.A.** 945, 1015, 1391, 1488, 1500, 1628, 1900, 2338, 2522, 2560, 3247 (*Lepidosperma inops*); 3844 (*Lepidosperma monticola*); 4016, 4105, 4354 (*Lepidosperma inops*); 4405, 4678 (*Lepidosperma monticola*), 5254 (*Lepidosperma inops*).

**Curtis, W.M.** *s.n.* 1 Jan. 1949 HO 135483 (*Lepidosperma inops*); *s.n.* 1 Jan. 1949 HO 135412, *s.n.* 1 Jan. 1949 HO 135486, *s.n.* 1 Jan. 1949 HO 135489, *s.n.* 7 Mar. 1949 HO 135487 (*Lepidosperma monticola*); *s.n.* 10 Sept. 1951 HO 135488, *s.n.* 3 Dec. 1972 HO 24548, *s.n.* 28 Oct. 1978 HO 135485, *s.n.* 28 Oct. 1978 HO 534984 (*Lepidosperma inops*).

**Davies, F.E.** 1241 (*Lepidosperma inops*).

**Dobson, A.T.** 77290 (*Lepidosperma monticola*).

**Duncan, F.** 2, 1120, *s.n.* 22 Mar. 1994 HO 317251, *s.n.* 1 Aug. 1995 HO 314343, *s.n.* 1 Aug. 1995 HO 314344, *s.n.* 31 Aug. 1995 HO 314345 (*Lepidosperma inops*).

**Griffith, S.J.** *s.n.* 20 Apr. 1993 NE (*Lepidosperma tortuosum*).

**Hodgon, J.** 391c NE, 415 NE (*Lepidosperma tortuosum*).

**Hosking, J.R.** 3041 NE (*Lepidosperma tortuosum*).

**Hunter, J.T.** 5068 NE (*Lepidosperma tortuosum*).

**Jackson, W.D.** *s.n.* 1 Apr. 1960 HO 24549, 30 Aug. 1983 HO 68636 (*Lepidosperma inops*).

**Jarman, S.J.** 157, 180, 235, 263, *s.n.* 16 Feb. 1978 HO 411020, *s.n.* 20 Dec 1978 HO31334, *s.n.* 13 Dec. 1979 HO 411752 (*Lepidosperma monticola*); *s.n.* 12 Aug. 1980 HO 319561 (*Lepidosperma inops*); *s.n.* 8 Feb. 1984 HO 412112, *s.n.* 21 Mar. 1984 HO 412037, *s.n.* 15 Jan.1985 HO 443183, *s.n.* 4 Mar. 1986 HO 412044, *s.n.* 12 Mar. 1986 HO 411078, *s.n.* 10 Apr. 1986 HO 412047, *s.n.* date unknown HO 411443 (*Lepidosperma monticola*).

**Klaphake, V.** *s.n.* 1 Jan. 1998 NE (*Lepidosperma tortuosum*)

**Moscal, A.** 214, 397, 409 (*Lepidosperma inops*); 1776, 1795, 4636 (*Lepidosperma monticola*); 6182 NE (*Lepidosperma inops*); 8022, 8039 (*Lepidosperma monticola*); 8040 (*Lepidosperma curtisiae*); 8254, 8285, 8291, 8363 (*Lepidosperma monticola*); 9441, 18031 NE (*Lepidosperma inops*); 19548, 28626 (*Lepidosperma monticola*).

**North, A.J.** *s.n.* 7 Oct. 1992 HO 132741, *s.n.* 7 Oct. 1992 HO 132754 (*Lepidosperma inops*); *s.n.* 8 Oct. 1996 HO 322622 NE (*Lepidosperma curtisiae*); *s.n.* 26 Sept. 2004 HO 530824 (*Lepidosperma inops*).

**Plunkett, G.T.** 94a, 94b, 95, 96, 98, 99, 100 (*Lepidosperma monticola*); 112, 115 (*Lepidosperma inops*) all NE.

**Rao, C.V.** *s.n.* 1 Jan. 1956 HO 135544 (*Lepidosperma inops*).

**Ratkowsky, D.A.** 545, 1082 NE, *s.n.* 1 Jan. 1974 HO 135490 (*Lepidosperma inops*); 8 Mar. 1974 HO 135415 (*Lepidosperma monticola*).

**Rodway, L.** *s.n.* 1 Mar. 1892 HO 26449 (*Lepidosperma inops*); *s.n.* 1 Mar. 1894 HO 26450 (*Lepidosperma monticola*).

**Smith, J.M.B.** 342 (*Lepidosperma monticola*).

**Williams, K.J.** *s.n.* 18 May 1987 HO 531508 (*Lepidosperma inops*).

**Wilson, K.L.** 6522, 6662 (*Lepidosperma inops*).

**Ziegeler, D.** 84, 114 (*Lepidosperma inops*).
